# Molecular Subtypes As Emerging Predictors of Clinicopathological Response to Neoadjuvant Chemotherapy (NACT) in Locally Advanced Breast Cancer (LABC): A Single-Centre Experience in Western India

**DOI:** 10.7759/cureus.25229

**Published:** 2022-05-22

**Authors:** Sarthak Sharma, Shaitan Singh Rathore, Vijay Verma, Murlidhar Kalyan, Narender Singh, Irshad Irshad

**Affiliations:** 1 General Surgery, Dr. Sampurnanand Medical College, Jodhpur, IND

**Keywords:** locally advanced breast cancer, neoadjuvant chemotherapy, pathological response, recist criteria, molecular subtypes

## Abstract

Introduction: Locally advanced breast cancer (LABC) is a subset of breast cancer characterized by the most advanced breast tumours in the absence of distant metastasis. Treatment of LABC has evolved from a single modality treatment to multimodality management. Neoadjuvant chemotherapy (NACT) is increasingly being used to treat patients with LABC. This study assessed tumour response after NACT using clinical changes, Response Evaluation Criteria in Solid Tumors (RECIST) criteria and pathological report.

Methodology: This study was a prospective as well as retrospective observational study carried out in the department of general surgery, Dr. Sampurnanand Medical College, Jodhpur. All the patients admitted with stage III (IIIA, IIIB, IIIC) were included in the study after obtaining approval from the institutional ethical committee. Clinical response was assessed by RECIST criteria (clinical complete response (cCR), clinical partial response (cPR), clinical progressive disease (cPD), and clinical stable disease (cSD)) and pathological response by histopathological report (pCR). Response of various molecular subtypes was noted.

Results: Among 31 patients included in the study, cCR observed in 22.58% cases, cPR observed in 61.29% cases while cPD and cSD seen in 3.22% and 12.90% cases, respectively. Pathological complete response (pCR) observed in 19.35% cases. Favourable response seen with human epidermal growth factor receptor 2 (HER2) overexpression (cCR = 50%, pCR = 37.50%) followed by triple negative (cCR = 25%, pCR = 25%) molecular subtypes.

Conclusions: It can be concluded that molecular subtype determination helps in deciding treatment protocol in patients with LABC with HER2 overexpression and triple-negative breast cancers having a better clinicopathological response to NACT than luminal subtypes. NACT results in downstaging of tumours, thus, help in achieving surgically clear margins and elimination of micrometastases which decreases the recurrence rates and morbidity/mortality of patients.

## Introduction

Breast cancer is the most commonly diagnosed cancer and the fifth cause of cancer deaths in the world, with an estimated 2.3 million cases and 685,000 deaths in 2020 [1]. Breast cancer in India accounts for 14% of all cancers in women. The incidence rates in India begin to rise in the early 30s and peak at ages 50-64 years [2].

Locally advanced breast cancer (LABC) represents an important and challenging problem. The US National Comprehensive Cancer Network (NCCN) describe LABC as American Joint Committee on Cancer (AJCC) stage III breast cancer that includes: tumours >5 cm (T3) with regional lymphadenopathy (N1-3), tumours of any size with chest wall or skin or both involvement (T4) irrespective of lymph node status, extensive lymph nodes involvement (matted axillary lymph nodes, or any of infra-clavicular, supra-clavicular, or internal mammary) [3].

Neoadjuvant chemotherapy (NACT) has become a primary option for patients with LABC [4]. The rationale for using NACT is to improve surgical options and to gain information on drug response by in-breast assessment. Furthermore, NACT provides the opportunity to discover predictive markers of chemotherapy [4]. The pathological complete response (pCR) is considered a biological marker for survival outcomes. The pCR is considered when there is complete eradication of locoregional disease [5,6].

We conducted this study in females with stage III LABC to evaluate the clinical response (Response Evaluation Criteria in Solid Tumors (RECIST) criteria) and pathological response shown by various molecular subtypes (luminal A, luminal B, human epidermal growth factor receptor 2 (HER2), and triple negative) to NACT.

## Materials and methods

The study was a prospective and retrospective observational study conducted in the department of general surgery, Mathura Das Mathur Hospital affiliated with Dr. Sampuranand Medical College, Jodhpur, Rajasthan. Institutional Ethics Committee, Dr. Sampuranand Medical College, Jodhpur issued approval SNMC /IEC/395 before the initiation of the study. Response rates were taken as the endpoint of the study.

After applying inclusion and exclusion criteria, 31 patients were identified for the study (Table 1). Initial staging workup included clinical examination supplemented by haematological (complete blood count), biochemical (renal and liver function tests), and radiological investigations (X-ray-chest, skeletal survey, and ultrasound abdomen). Computed tomography and magnetic resonance imaging were done wherever indicated. Immunohistochemistry was obtained for all the patients.

**Table 1:** Inclusion and exclusion criteria AJCC: American Joint Committee on Cancer; TNM: tumour, node, metastasis

Inclusion Criteria	Exclusion Criteria
Previously untreated female patients with bi-dimensionally palpable and measurable primary breast cancer stage III (according to AJCC TNM staging system, 8^th^ edition) diagnosed by core needle biopsy.	Patient not willing for study;
	All cases diagnosed as benign disease of breast;
	Breast carcinoma patients not lying in the definition of locally advanced breast cancer (LABC) clinically and/or radiologically;
	All patients with presence of distant metastasis proved on clinical examination/investigations.

Patients were subjected to anthracycline-based NACT after the initial assessment was completed. Chemotherapy consisted of Adriamycin 60 mg/m^2^ body surface area (BSA) IV over 3 hours and cyclophosphamide 600 mg/m^2^ BSA IV over 3 hours. All patients received four cycles of NACT (q21 days). All the patients were subjected to modified radical mastectomy (MRM) after NACT and specimens were sent for histopathological examination (HPE).

Evaluation of neoadjuvant chemotherapy response

Clinical response was assessed by RECIST criteria (Table 2). 

**Table 2:** RECIST criteria RECIST: Response Evaluation Criteria in Solid Tumors

RECIST Criteria	
Clinical complete response (cCR)	No palpable tumour in the breast and axilla
Clinical partial response (cPR)	≥30% reduction in the maximum dimension of the tumour mass
Clinical progressive disease (cPD)	≥20% increase in the maximum dimension of tumour mass
Clinical stable disease (cSD)	When the change does not meet any of the other criteria

Pathological response was assessed by histopathology. pCR implied no residual invasive breast cancer in the histopathology specimen of breast and axillary lymph nodes (ypT0/ypTisN0). All comparisons of categorical variables were performed with the chi-square test while continuous variables were compared using the t-test. All the results are presented with p-values.

## Results

The median age of the patients at the time of diagnosis was 49 years (range: 25-79 years). The maximum number of patients were in the age group of 41-50 years (41.93%) followed by 51-60 years (25.80%). 61.29% were premenopausal at the time of diagnosis. The clinical characteristics are listed in Table 3.

**Table 3:** Clinicopathological characteristics of the patients and molecular subtypes HER2: human epidermal growth factor receptor 2

Characteristics	Number of patients (n=31)
Age (years), median (range)	49 (25-79)
Menopausal status	
Premenopausal	19 (61.29%)
Post-menopausal	12 (38.71%)
Lump laterality	
Right	18 (58.06%)
Left	13 (41.94%)
Lump size	
<5 cm	2 (6.45%)
>5 cm	29 (93.54%)
Nodal status	
Negative	0
Positive	31 (100%)
Clinical stage	
III A	18 (58.06%)
III B	11 (35.48%)
III C	2 (6.45%)
Histological type	
Invasive ductal carcinoma	28 (90.32%)
Invasive lobular carcinoma	1 (3.23%)
Others	2 (6.45%)
Molecular classification	
Luminal A	10 (32.26%)
Luminal B	5 (16.13%)
HER2 overexpression	8 (25.81%)
Triple negative	8 (25.81%)

The lump was the most consistent presenting symptom and was present in 100% of the cases. The upper outer quadrant (58.06%) was the most commonly involved quadrant and the least incidence was found in the lower inner quadrant (3.23%). The tumour size ranged from 4 to 12.5 cm with a mean size of 6.11 cm. All the patients were lymph node positive at the time of presentation. 90.32% patients had invasive ductal carcinoma (IDC).

Clinical and pathological response rates according to molecular subtypes

A clinical response was observed in 83.87% of the cases subjected to NACT as assessed by RECIST criteria with clinical complete response (cCR) noted in 22.58% cases. 51.61% cases became clinically lymph node-negative following NACT (p value <0.0001) and clinical downstaging was observed in 67.74% cases (p value <0.0001). The response shown by various molecular subtypes is listed in Table 4.

**Table 4:** Clinical Response Rates in Molecular Subtypes cCR: clinical complete response; cPR: clinical partial response; cPD: clinical progressive disease; cSD: clinical stable disease; HER2: human epidermal growth factor receptor 2

Subtype	cCR	cPR	cPD	cSD
Luminal A	1 (10.00%)	7 (70.00%)	1 (10.00%)	1 (10.00%)
Luminal B	0 (0.00%)	4 (80.00%)	0 (0.00%)	1 (20.00%)
HER2 overexpression	4 (50.00%)	3 (37.50%)	0 (0.00%)	1 (12.50%)
Triple Negative	2 (25.00%)	5 (62.50%)	0 (0.00%)	1 (12.50%)
Total	7 (22.58%)	19 (61.29%)	1 (3.22%)	4 (12.90%)

cCR was observed in a maximum of 50% of the cases of HER2 overexpression followed by 25% of cases of triple-negative breast cancer. No cCR was noted in luminal B subtype. pCR was observed in 19.35% cases included in the study. The rates of pCR in different molecular subtypes are depicted in Table 5.

**Table 5:** Pathological response rates in molecular subtypes HER2: human epidermal growth factor receptor 2

Subtype	pCR	No pCR
Luminal A	1 (10.00%)	9 (90.00%)
Luminal B	0 (0.00%)	5 (100%)
HER2 overexpression	3 (37.50%)	5 (62.50%)
Triple negative	2 (25.00%)	6 (75.00%)

Maximum (37.50%) pCR was observed in the HER2 overexpression subtype followed by 25% in the triple-negative subtype. No pCR was observed in luminal B subtype. An observation regarding the common side-effects of chemotherapeutic drugs was made. Alopecia was the most common side effect which was present in 100% cases followed by anaemia (58.06%), nausea and vomiting (45.16%), and neutropenia (38.71%).

## Discussion

LABC poses a challenge to the treating surgeon in terms of choosing the appropriate treatment for his/her patient. With advances in cancer management, especially breast cancer, with the availability of systemic and hormonal therapies and breast conservation surgery, it has become important to individualize patient’s treatment.

There has been a constant debate regarding NACT’s role in LABC. Neoadjuvant chemotherapy leads to cytoreduction of the primary tumour and regional lymph nodes allowing breast conservation in selected cases. An additional benefit of this technique is, potentially, eradication of distant microscopic metastases, the presence of which is the most common cause of relapse. Moreover, early application of chemotherapy may also guide postoperative therapeutic options by evaluating a tumour’s chemosensitivity. However, it is feared that delaying the initiation of definitive therapy for operable LABC, if the tumour burden is large, might lead to the development of drug resistance and subsequently systemic metastases. Besides, toxicity from early use of chemotherapy might render a patient a nonsurgical candidate [7].

In our study, 31 patients were included who had a diagnosis of stage III LABC at the time of presentation. The maximum number of patients were in the age group of 41-50 years (41.93%) followed by 51-60 years (25.80%) and median age was 49 years. This age distribution of patients was similar to previous studies [8,9,10]. A lump in the breast was the commonest symptom noticed in 100% cases in our study which was similar to other studies [11,12,13]. Upper outer quadrant (58.06%) was the most commonly involved quadrant and the least incidence was found in lower inner quadrant (3.23%). The tumour size ranged from 4 to 12.5 cm with a mean size of 6.11 cm. All the patients were lymph node positive at the time of presentation. 90.32% of patients had IDC.

Gene expression studies have identified distinct molecular subtypes of breast cancer with a different prognoses [14,15,16]. It is also believed that various subtypes of breast cancer show different sensitivities to systemic chemotherapy. In the present study, we used molecular classification and divided patients into four subtypes: luminal A, luminal B, HER2 overexpression, and triple-negative and assessed the response of each subtype to anthracycline-based NACT.

Clinical response was assessed by calculating the percentage decrease in the volume of the tumour. The overall response to NACT in this study was 83.87% with partial response seen in 61.29% cases and complete response seen in 22.58% cases which was similar to previous studies [17,18]. 51.61% of cases became clinically lymph node-negative following NACT (p value <0.0001).

cCR was seen in maximum 50% cases of HER2 overexpression followed by 25% cases of triple-negative breast cancer while luminal types had low cCR rates with no cCR seen in luminal B subtype. Clinical partial response (cPR) was observed in 70% of luminal A, 80% of luminal B, 62.50% of triple negative, and 37.50% of HER2 overexpression subtype.

The pathological response was assessed by the HPE of the MRM specimen. pCR was noted in 19.35% cases. Among molecular subtypes, maximum of 37.50% pCR was observed in HER2 overexpression followed by 25% cases of triple-negative subtype. No pCR was observed in luminal B subtype. Gentile et al. in their study observed pCR in 48% cases of HER2 overexpression and 23% cases of triple-negative breast cancer [19]. Luangdilok et al. and Subbiah et al. also showed that HER2 overexpression and triple-negative breast cancer subtypes had more pCR as compared to luminal subtypes [20,21].

There are multiple potential reasons that the response to chemotherapy differed by subtype. The triple-negative and HER2 overexpression breast cancers are characterized by the high expression of the proliferation cluster of genes [14,15]. Furthermore, the triple-negative tumour is basically estrogen/progesterone (ER/PR) negative, and evidence suggests that the negative hormone receptor status is one of the strongest predictive markers associated with the higher likelihood of pCR to NACT [22,23,24].

Chemotherapy has got its own complications and incidence wise alopecia, anaemia, nausea and vomiting are the commonest. In the present study, alopecia was the most common side effect which was present in 100% cases followed by anaemia (58.06%), nausea and vomiting (45.16%), and neutropenia (38.71%).

Limitations of study: The study was conducted in a single institution and had a small sample size.

## Conclusions

LABC is a common presentation of carcinoma breast in India and the role of NACT has been debated for a long time now. In our study, clinical response (complete plus partial) resulting in a decrease in tumour size was observed in more than 80% cases. Pathological disappearance of the invasive tumour was noted in approximately 20% cases.

Assessment of response of molecular subtypes to NACT suggests that HER2 overexpression and triple-negative breast cancers have better clinicopathological response than luminal subtypes. Hence, molecular subtype determination helps in deciding treatment protocol in patients with LABC. NACT results in downstaging of tumours, thus, help in achieving surgically clear margins and elimination of micrometastases which decreases the recurrence rates and morbidity/mortality of patients.
